# Assessment of community readiness to address malnutrition in rural southwest Guatemala

**DOI:** 10.1002/puh2.164

**Published:** 2024-04-11

**Authors:** Annika M. Weber, Roberto Delgado‐Zapata, Melissa Fineman, Andrea Jimenez‐Zambrano, Brigitte A. Pfluger, Maureen Cunningham, Diva M. Calvimontes, Elizabeth P. Ryan, Molly M. Lamb

**Affiliations:** ^1^ Department of Food Science and Human Nutrition Colorado State University Fort Collins Colorado USA; ^2^ Center for Global Health Colorado School of Public Health Aurora Colorado USA; ^3^ Department of Community & Behavioral Health Colorado School of Public Health Aurora Colorado USA; ^4^ Department of Pediatrics University of Colorado School of Medicine Aurora Colorado USA; ^5^ Nutrition and Health Sciences Laney Graduate School Emory University Atlanta Georgia USA; ^6^ Center for Human Development Fundación Para la Salud Integral de los Guatemaltecos, FUNSALUD, Coatepeque Quetzaltenango Guatemala; ^7^ Department of Environmental and Radiological Health Sciences Colorado State University Fort Collins Colorado USA; ^8^ Department of Epidemiology Colorado School of Public Health Aurora Colorado USA

**Keywords:** children, community readiness, Guatemala, malnutrition, nutrition, undernutrition

## Abstract

**Background:**

Malnutrition is prevalent throughout southwest Guatemala, where >40% of children suffer from chronic undernutrition. Evidence supports that assessing a community's awareness and readiness to address malnutrition is a critical first step in improving the success of a nutrition intervention program. The objective of this study was to apply the community readiness model (CRM) to assess community readiness to address childhood malnutrition in a rural southwest region of Guatemala.

**Methods:**

Thirteen key respondents of varied social roles and demographics residing in the region were interviewed. Interview questions related to addressing malnutrition were from the following predefined dimensions: Community Efforts, Community Knowledge of Efforts, Leadership, Community Climate, Community Knowledge, and Resources for Efforts. Interview recordings and notes were analyzed and scored according to the CRM guidelines, and a standardized analysis was conducted.

**Results:**

The overall community readiness score was 4.26 (preplanning: awareness of the issue). Community Efforts had a total score of 5 (Preparation: preparing to take action on the issue). Community Knowledge of Efforts, Community Climate, Community Knowledge, and Resources for Efforts Dimensions each had a total score of 4 (Preplanning: awareness of the issue). The overall score for the Leadership dimension was 2 (Denial/resistance: belief that the problem does not exist within the community). These scores demonstrate clear recognition for action to address childhood malnutrition as a problem. However, efforts to combat childhood malnutrition are not yet focused nor detailed for community action.

**Conclusions:**

This rural southwest region of Guatemala recognizes that childhood malnutrition is a problem. However, efforts to address malnutrition are not yet focused or detailed enough to have measurable impact in addressing this issue. For the region to advance the stage of community readiness, it is essential to enhance knowledge of dietary strategies aimed at improving nutrition for children and increase engagement from local leadership.

## Graphical abstract

Understanding a community's readiness to address malnutrition is critical to the design and success of future intervention programs. Using the community readiness model, we determined that a rural southwest region of Guatemala was at the pre‐planning stage, indicating a clear recognition of need for action to address childhood malnutrition.

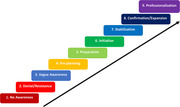

## INTRODUCTION

Globally, an estimated 45 million children under 5 years of age experience wasting, and 148 million children are stunted [1]. The prevalence of stunting in Latin America and the Caribbean has declined in the last few years (16.9% in 2000–9.6% in 2017) [2]. Guatemala, however, consistently has the highest rate of stunting within Central America and is the sixth highest worldwide [3]. Stunting affects almost half of all children under 5 years of age, reaches close to 70% in some departments [1, 4, 5], and disproportionately affects rural Guatemalan communities [5]. Latin America and the Caribbean continue to face complex issues of malnutrition and globally are ranked the region with the highest cost for a healthy diet [6]. COVID‐19 has exacerbated food insecurity, undernutrition, and stunting worldwide, including in Guatemala [7]. A recent survey in the rural southwest region of Guatemala revealed that, during the COVID‐19 pandemic, households experienced dramatic increases in mild (from 56% to 91%), moderate (from 34% to 87%), and severe food insecurity (from 11% to 20%) [7, 8].

The consequences of childhood malnutrition can be life‐long. Inadequate nutrition early in life increases susceptibility to diarrheal and respiratory diseases as well as increases the risk for morbidity, mortality, and delayed cognitive development [9, 10, 11]. These conditions can subsequently impact academic achievement, resulting in fewer years of schooling, earlier childbearing, and reduced lifetime productivity [12, 13].

A strong foundational understanding of a community's needs and willingness to participate in improving nutrition is central to building sustainable malnutrition prevention programs and treatment strategies. The prevention and treatment of malnutrition involves multisector stakeholder engagement at local, regional, and national levels. Government participation and community engagement require local leaders, healthcare workers, and families to inform program design and implementation, all of which can maximize malnutrition reduction efforts suitable for a given population [14, 15]. Community assessments are intended to identify feasible approaches to address an issue that result in actionable measures. The community readiness model (CRM) is an open‐access tool developed by the Tri‐Ethnic Center for Prevention Research at Colorado State University [16]. The CRM is designed to assess a community's degree of readiness to address a specific issue. This tool has been essential in the successful development and implementation of programs related to a wide range of topics [17, 18, 19, 20, 21]. The objective of this study was to apply the CRM to assess current awareness and readiness to address the complex issues of childhood malnutrition in a rural region of southwest Guatemala that suffers from high rates of food insecurity and childhood malnutrition [22, 23]. The findings of this formative research will guide community‐driven nutrition programs aimed toward the prevention and treatment of malnutrition as well as improving local food security.

## METHODS

This qualitative study, based on the CRM, was implemented to obtain a comprehensive view of the community's awareness and readiness to take action to address childhood malnutrition in a rural southwest region of Guatemala. The region, known as the southwest Trifinio region, intersects the San Marcos, Quetzaltenango, and Retalhuleu departments. In September 2021, key respondents were selected by a local study coordinator from towns within this region and consisted of Chiquirines, Los Encuentros, Barillas, Valle Lirio, La Blanca, Palmar II, and Coatepeque. A convenience sample of 13 community stakeholders participated in this semi‐structured interview. The study team defined the desired socioeconomic, political, and professional roles for diverse key respondents residing in the region, based on those the problem impacts directly and those with decision‐making power. The diverse community positions and roles represented for the interviews were the following: doctor; nurse; or other healthcare worker; educator; community advisory board member (CAB; who represents the community interests and advises local research activities); mayor or other political leader; mother of young child(ren); grandmother of young grandchild(ren); religious leader; agricultural worker; rice mill operator/owner; and local merchant.

### Data collection

From September 27 to October 1, 2021, study team members conducted 13 interviews with key respondents in their homes or workplaces. The interview questionnaire was created by the research team based on the CRM and was reviewed and adapted with feedback from two community members who were not part of the study. Written informed consent for the interviews and audio recordings used for analysis were obtained before the start of each interview. Interviews were conducted in person by a native Spanish speaker from the community and ranged between 25 and 60 min. The CRM assessment interview form (see Supplementary materials) was used to guide the interviews. The six dimensions of the CRM assessment are (A) Community Efforts (to what degrees are existing efforts in place); (B) Community Knowledge of Efforts (to what degrees do community members know about existing efforts); (C) Leadership (how involved are community leaders in the issue); (D) Community Climate (how do community members view the issue); (E) Community Knowledge about the Issue (to what degrees are community members aware of the issue); and (F) Resources for Efforts (to what degrees are resources available to combat the issue) [17, 18]. Interview questions spanned across the six dimensions and were grouped accordingly on the interview form (see Supplementary materials).

For each interview, hand‐written notes were taken by the main interviewer in Spanish, and by a second Spanish‐speaking study team member. Twelve of the interviews were audio‐recorded using a handheld recording device. One participant did not consent to the audio recording, and this interview had detailed note‐taking to standardize the capture of all the responses. All 13 interviewed respondents were reimbursed with a prepaid phone card for their time and participation.

### Community readiness model analysis of interviews

**FIGURE 1:**
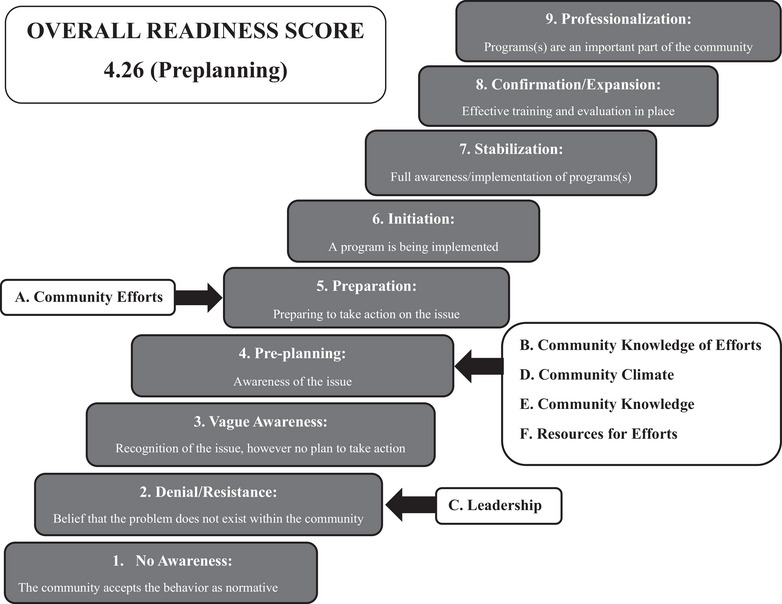
Dimensions and overall readiness scores plotted to the stages of the community readiness model assessment. Overall readiness is at the preplanning stage for the rural southwest Guatemala region. *Source*: Adapted from Ref. [18].

Interview audio recordings and accompanying hand‐written notes were scored by a native Guatemalan Spanish speaker who has lived and worked in the region and is familiar with the local dialect. This individual did not participate in and was not present for the in‐person interviews. Each interview was scored independently. To score the interviews, the scorer read the facilitator's notes, listened to the interview recording, and used a clean scoring sheet to document their comments, impressions, and qualifying statements about the interviewee's answers regarding the six dimensions. After listening to and annotating the completed interview, the scorer reviewed and assigned a score between 1 and 9 (1 = no awareness, 9 = there is professionalization and community ownership of the problem, Figure 1) for all six dimensions using the CRM assessment scoring algorithm of the Community Toolbox [14]. Scores were averaged within each dimension to determine total dimension scores. To determine the community's overall readiness score, an average of the six‐dimension scores was calculated. In addition to the questions related to assessing the six dimensions of the CRM, nine additional questions on food behavior were included as an additional analysis (not part of scored CRM analysis; Supplementary materials). All analyses were completed using Microsoft Excel and GraphPad Prism 9.5.1.

### Ethics

The study was reviewed and approved by the Colorado Multiple Institutional Review Board (COMIRB Protocol 21‐3227); Colorado State University IRB cedes to COMIRB (CSU IRB protocol ID 2511). This protocol was also approved by the Comite Independiente de Etica y Investigacion Cientifica (CIEIC K'awil), the local Ethics Review Board of Guatemala.

## RESULTS

### Community readiness to address childhood malnutrition

**FIGURE 2:**
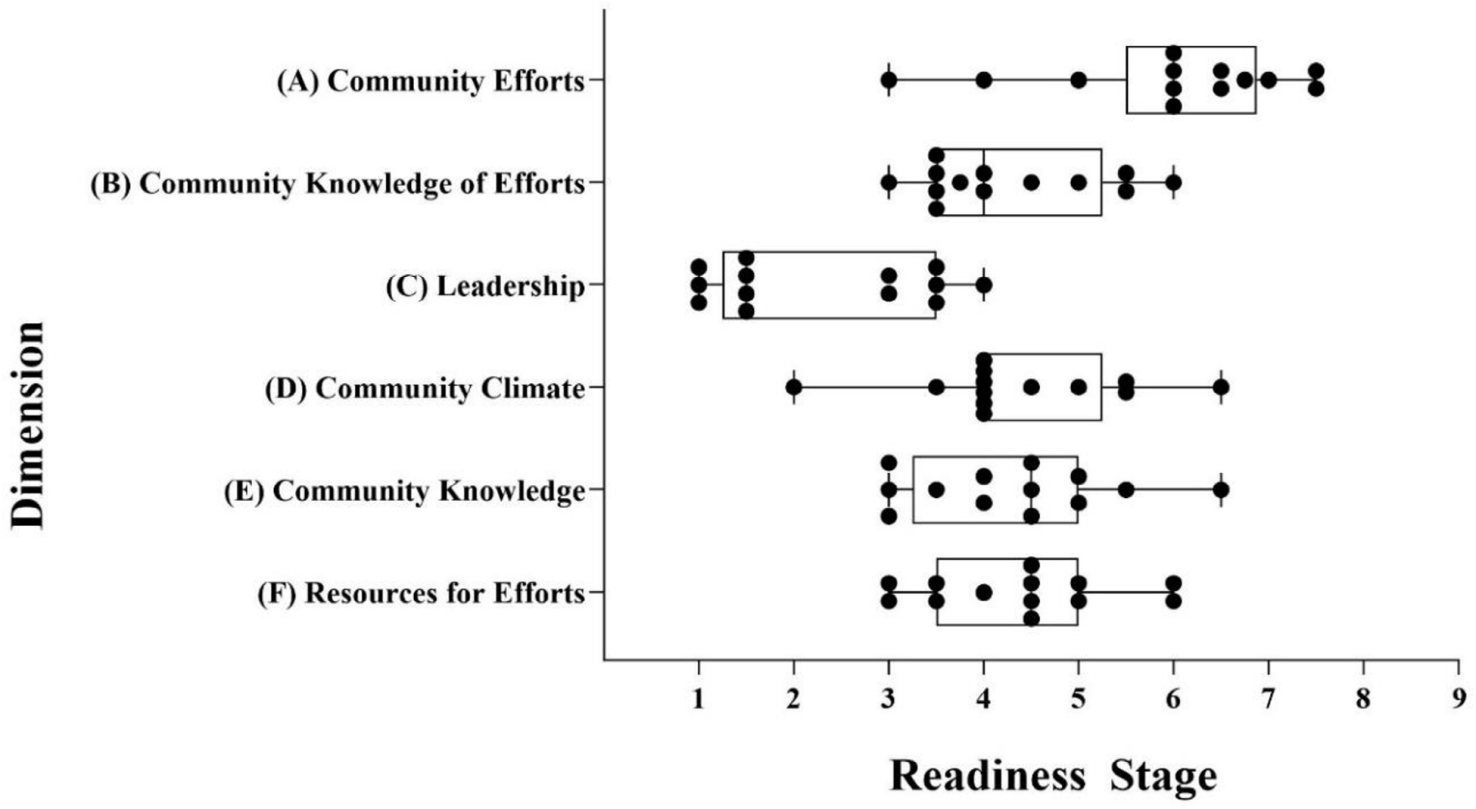
Community readiness stages for all six dimensions related to addressing malnutrition in the rural southwest region of Guatemala (median, minimum, and maximum scores). Scales shown represent 1 = no awareness, to 9 = there is professionalization and community ownership of the problem. Data points represent individual responses.

The key informants were 46% male, lived in seven different communities in the region, and held one of the desired social/professional roles in their community. See Table 1 for demographic details of the key respondents. The overall community readiness score was 4.26 (SD 1.52), which is the preplanning stage of readiness to address childhood malnutrition. Community Efforts had a total score of 5 (Preparation: preparing to take action on the issue), though individual respondent scores ranged from 3 (Vague Awareness: recognition of the issue, however no plans to take action) to 7.5 (Stabilization: full awareness/implementation of programs). Community Knowledge of Efforts, Community Climate, Community Knowledge, and Resources for Efforts Dimensions each had a total score of 4 (preplanning: awareness of the issue). The overall score for the Leadership dimension was 2 (Denial/resistance: belief that the problem does not exist within the community), and no interviewee had a score of more than 4 for this dimension (Figures 1 and 2).

**TABLE 1:** Key respondent characteristics of community members from the rural southwest Guatemala region that were interviewed in this community readiness model assessment.

Characteristic	Number of key respondents (*n* = 13) *n* (%)
Age range, years	
20–34	5 (38)
35–44	3 (23)
45–54	3 (23)
55–64	0 (0)
65+	2 (15)
Male	6 (46)
Community	
Coatepeque	2 (15)
Los Encuentros	2 (15)
Chiquirines	2 (15)
Valle Lirio	2 (15)
La Blanca	1 (8)
Barillas	2 (15)
Palmar II	1 (8)
Did not say	1 (8)
Social role/profession	
Community leader (assistant mayor, advisory board)	2 (15)
Health care worker	3 (23)
Educator (teacher/principal)	2 (15)
Religious leader	1 (8)
Merchant	1 (8)
Agricultural worker	1 (8)
Mother/grandmother	3 (23)

**FIGURE 3:**
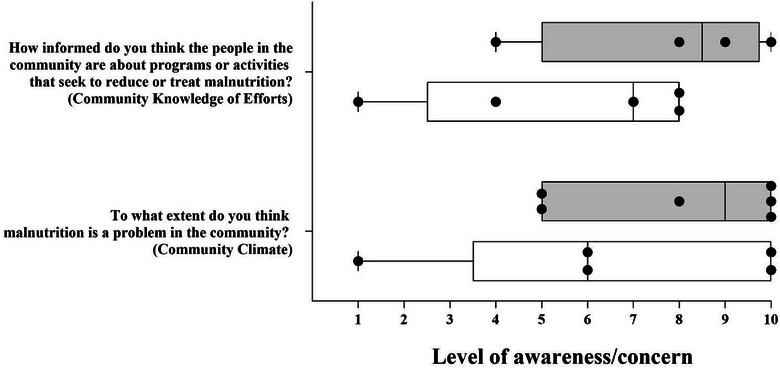
Gender differences in Community Knowledge of Efforts and Community Climate. Numerically scaled responses from 1 to 10 (1 = “no awareness/concern at all” and 10 = “very aware/concerned”) to two specific malnutrition questions (median, minimum, and maximum scores). Data points represent participant scores for those who provided responses.

Within the Community Knowledge of Efforts dimension of the questionnaire, key respondents were asked to rank on a numerical scale of 1–10 (1 = “no awareness at all” and 10 = “very aware”) how informed they believe the community is about local programs and activities related to combating childhood malnutrition. The rankings for this question by key respondents who provided numeric responses to these two questions are illustrated in Figure 3. All female respondents gave a ranking of above 4, one of which gave a ranking of 10. Male rankings for this question ranged from 1–8.

Respondents were also asked to rank on a numerical scale of 1–10 (1 = “not a problem” and 10 = “it is a big problem”) their belief that childhood malnutrition is a problem in the region as part of the Community Climate dimension. Of the responses, eight gave numerical responses, two females, and one male did not give numerical responses but instead verbally responded that malnutrition is “a big problem” in the region, and two did not provide a response. All females gave a rank of 5 or more, indicating a stronger belief among female respondents that malnutrition is an issue in the region. One male indicated a rank of 1, and one male and one female both ranked this question as 10 (Figure 3).

Although rankings from these questions related to Community Knowledge of Efforts and Community Climate suggest that there is awareness and concern about malnutrition prevalence in the community, the scores spanned the full range of 1–10 for each question, indicating a full spectrum of attitudes and opinions. A potential explanation for the spectrum of responses was how knowledge and awareness are framed by family experiences with malnutrition. For example, those who expressed little knowledge concerning the prevalence and treatment of malnutrition in the community disclosed that their families had never faced or experienced malnutrition problems (Table 2).

**TABLE 2:** Selected comments from interviews with community members in the rural southwest region of Guatemala.

**Community efforts**—Who in this community is trying to do something to reduce child malnutrition? *“…the little hospital sometimes sends someone to visit the families with small kids” (male, CAB member)* *“Nurses, teachers, and cocodes (for example community chief) … we need to include cocodes because they know the location of each family and which families might need help and which kids specifically” (male, nutritionist)*
**Community knowledge of efforts—**How informed do you think the people in the community are about programs or activities that seek to reduce or treat malnutrition? *“Only the families that have malnourished children are aware of malnutrition. But those that don't have malnourished children are not.” (male, mayor's assistant*) *“The vast majority of people are unaware [of malnutrition programs]” (female, primary school teacher)*
**Leadership—**What are community leaders or people doing to reduce malnutrition? *“[the leaders of the community] give us some groceries … beans, rice, and cornmeal” (female, housewife)* *“What leaders are you talking about? I don't know of leaders caring for their community” (female, grandma)* *“The leaders provide counselling, advice, and notify the health center or refer the families to visit the health center” (male, nutritionist)*
**Community climate—**Do you think that addressing malnutrition is very important for people in your community? Do communities support addressing the problem of malnutrition? If yes, do you think they would actively support or participate in these programs or activities? *“Quite. A community that is healthy … is going to be a capable community that is going to sustain itself, develop and grow. That is what everyone wants, that their place where they live is in good condition.” (male, nutritionist)* *“Quite a lot. because [addressing malnutrition] in children more than anything can reduce the problem of illiteracy and supports good immune development.” (female, Nurse)* *“It's very important. Because we want in the future to have good children, because they are the hope of our country. We want good and educated people. Although we are peasants, but literate.” (Male, CAB Member)* *“Yes, they support [the problem] because they are already aware of how important it is to combat malnutrition. They know that a healthy population is much cheaper to maintain and sustain in the future due to chronic diseases that may occur in the future” (male, doctor)* *“If the mothers … have love for their children and if they have any of their children malnourished, they have to worry.” (female, housewife)*
**Community knowledge** —In general, how much do people in the community know about malnutrition? *“They know that it is not good to have a malnourished child. The problem is that they do not have the financial resources” (male, doctor)* *“They know that a malnourished child is going to be sick. But they don't know the signs and symptoms and what it means to have malnutrition and grow up malnourished.” (male, nutritionist)*
**Resources/barriers**—What are the primary barriers or difficulties (or obstacles) to address this issue in the community? *“Everything comes from the fact that people do not collaborate. You can have the best interest … People just want money, but they don't come [to the health post for weights and heights]” (female, nurse)* *‘‘Parents do not collaborate in ensuring the well‐being of their children … Sometimes parents let their children be sick … That should not happen because every parent should make an effort to ensure that their children grow up well. (male, religious leader)*

Abbreviation: CAB, community advisory board.

### Community food behaviors

In the additional questions related to food behavior, such as “who is in charge of purchasing food in the family?” most respondents named the “mother,” “wife,” or “woman”, whereas the “father,” “husband,” or “son” were responsible for planting/harvesting food and providing the household income. When assigning household responsibilities, many singled out mothers as responsible for the nutritional status of the child and for identifying and treating the problem of malnutrition.

A topic that permeated many of the food behavior questions was the notion that the COVID‐19 pandemic had greatly influenced employment and food prices in the community. Economic instability and inconsistent work availability were identified as the most important and constricting factors of food‐related decisions. Families stated that their food choice is dictated by those that are more available and affordable. Many specifically mentioned how the rise in the cost of the basic food basket (i.e., the most economical food products available) means that meat is now a luxury item for many families.

When asked about food behaviors related to the introduction of new foods or ingredients into the household diet, the responses indicated openness and interest in trying new items. Respondents referenced past community gardening and food programs that energized community members. Although optimistic about the community's willingness to try new foods, palatability, affordability, and knowledge of nutritional benefits were named critical for their full and long‐lasting integration into the local diet. See Table 2 for examples of sentiments expressed by community members related to the dimensions of the assessment.

As part of this supplemental section of the interview, respondents were also asked who should oversee the improvement of malnutrition in the community. Many interviewees/respondents specifically named “community leaders,” “mayors,” “educators,” “the government,” “the president,” and “Cocodes” (Community Development Councils ‐Consejos Comunitarios de Desarrollo Urbano y Rural). One suggested approach that emerged from the interviews was to increase the involvement of the local leadership, engaging not only politicians in these issues but also doctors, medical personnel, religious leaders, and educators. Another outcome proposed from the interviews was to publicly raise awareness and to bring attention to local impacts of malnutrition via radio announcements, thereby increasing community outreach for existing malnutrition programs and encouraging a community‐led group that engages leaders to develop malnutrition reduction strategies.

## DISCUSSION

The community readiness assessment indicated that this rural southwest region of Guatemala is in the pre‐planning stage for addressing malnutrition. This indicates that there is community‐wide recognition that childhood malnutrition is an issue in the region. However, although there is awareness of groups addressing nutrition and food security, our analysis identified that efforts to address malnutrition are not yet focused or detailed enough to have sufficient measurable impact in addressing the complexity of the problem.

Leadership scored the lowest of the six dimensions from the CRM, indicating that more directed efforts by community leaders are required for the community to move to the next level of readiness. The implementation of small forums led by community leaders to gather information on household concerns and to identify useful programs is also suggested. Interestingly, Community Knowledge of Efforts scored lower than the Community Efforts dimension. This suggests that although respondents were aware of food security and malnutrition prevention programs, they did not believe that their fellow community members were aware of these programs. This further supports the notion that there is discordance within the community, and there is a need for strategic and actionable steps forward to address this problem. A systematic scoping review on chronic malnutrition in Guatemala confirmed a lack of multilevel nutrition interventions, and a need for widespread efforts to address structural issues of childhood nutrition [24]. Some previous concentrated efforts in Guatemala to address malnutrition have had positive impacts, such as food security initiatives [25, 26], food fortification programs [27], and longitudinal nutrition intervention studies [28, 29]. However, these programs require continued engagement from leadership to implement programs as well as raise awareness of initiatives in place.

Awareness and concern of malnutrition in the community between females and males also displayed discrepancies, whereby females generally responded with more awareness of the issue of malnutrition in their community. This likely stems from defined gender roles in the area noted by references to the “women” or “mother” with responsibilities that pertain to the nutritional status of the child and the “male” or “father” responsible for family income and crop production/harvest. Similar divergences in awareness of childhood malnutrition were found between females and males in a community readiness study in Tehran, Iran. Here, after an intervention focused on improving readiness to address childhood obesity in a community, females had a statistically significant greater overall community readiness score than males [30]. These gender differences are crucial to consider when facilitating future community change activities related to addressing childhood malnutrition.

Furthermore, the additional questions found that respondents were likely to make food choices for their family based on cost and should be considered for future food security and malnutrition prevention programs. Key respondents also answered that they believed their community members would be interested in incorporating new foods into their diets if it was palatable, affordable, and added nutritional benefit to their local diet. This community perspective and openness indicate that this is a promising region for the implementation of novel nutrition programs aimed toward the prevention and treatment of malnutrition. This notion was confirmed by the high compliance and acceptance in a recent rice bran dietary intervention trial in the southwest region of Guatemala communities [31]. A similar CRM to identify readiness to address childhood diarrheal disease and food security issues in Dioro, Mali was applied. Here, they found that there was community enthusiasm around the incorporation of additional ingredients to traditional foods to improve nutrition and health [20]. Since this initial CRM assessment in Mali, an effective dietary intervention study was held in this community to prevent childhood malnutrition and decrease diarrhea incidence [32]. This demonstrates the importance of perspective gathered from community readiness assessments for the success of novel nutrition initiatives.

Study limitations include that there was only a single scorer for the interviews. Furthermore, this model seeks to quantify a qualitative dataset in a standardized manner, and as such this scoring system contains some assumptions by the single scorer. However, this scorer did not participate in the interviews and so remained objective. Furthermore, this CRM evaluation is a snapshot in time, and the community readiness may fluctuate. A strength of this study includes the structured CRM approach to assess community readiness. Moreover, as the interviews were all conducted within the same week, concern of temporary trends was avoided. The CRM typically requires four to six key respondents to complete an interview within a community. In this CRM assessment, we expanded the interview set to 13 respondents in various roles and professions within the region, giving a robust representation of the community and adding to the strength of responses obtained to each question and integrated for analysis. In addition, the entire process of designing, conducting, and analyzing the interviews was conducted by native Spanish speakers reducing information lost in translation.

## CONCLUSIONS

The results from this CRM assessment demonstrate that community members in this rural southwest region of Guatemala recognize childhood malnutrition as an important issue. However, although some are aware of local efforts, there is limited detailed knowledge of the local malnutrition prevention programs in place. These results enabled us to identify key barriers to effectively address malnutrition in this rural region. Activities to advance community readiness beyond the preplanning stage are necessary. The next steps identified include organizing community collaboration, building knowledge of dietary strategies related to improving nutrition for children, as well as increasing the engagement of local leaders. These may include but are not limited to dietary strategies to improve food access and nutrition for children. This CRM was essential in the formative research of baseline community readiness in this region and provides important community feedback on topic areas where targeted and information‐directed efforts can be made. Such findings are critical for the development of future dietary intervention studies and ongoing malnutrition treatment programs in rural southwest Guatemala.

## AUTHOR CONTRIBUTIONS

Study conception and design were done by Roberto Delgado‐Zapata, Diva M. Calvimontes, Andrea Jimenez‐Zambrano, Maureen Cunningham, Elizabeth P. Ryan, and Molly M. Lamb. Data collection was completed by Roberto Delgado‐Zapata, Diva M. Calvimontes, Annika M. Weber, and Molly M. Lamb. Data analysis was performed by Melissa Fineman. Preparation of the manuscript was led by Molly M. Lamb, Annika M. Weber, Roberto Delgado‐Zapata, Diva M. Calvimontes, and Elizabeth P. Ryan with critical revision led by Brigitte A. Pfluger and all other authors. All authors are accountable for all aspects of the work and have reviewed and approved the final version.

## CONFLICT OF INTEREST STATEMENT

The authors declare no conflicts of interest.

## ETHICS STATEMENT

The study was reviewed and approved by the Colorado Multiple Institutional Review Board (COMIRB Protocol 21‐3227); Colorado State University IRB cedes to COMIRB (CSU IRB protocol ID 2511). This protocol was also approved by the Comite Independiente de Etica y Investigacion Cientifica (CIEIC K'awil), the local Ethics Review Board of Guatemala.

## Supporting information

Supporting Information

## Data Availability

The data that support the findings of this study are available from the corresponding author upon reasonable request.
