# Supplementary material for: Assessment of community readiness to address malnutrition in rural southwest Guatemala
Source: Public Health Chall. 2024 Apr 11;3(2):e164. doi: 10.1002/puh2.164 (PMC11643428; doi:10.1002/puh2.164)
Supplement: Supplementary file 1 — Supporting Information [file PUH2-3-e164-s001.docx]

**Supplementary Documents: Community Readiness Assessment Interview form (in English)**

**INTERVIEW GUIDE**

**Subject ID: ________________________________**

**Interviewer’s name: ____________________________**

**Date of interview: __________________________**

**Interview questions to assess improving food security through dietary intervention of rice bran. (Community Readiness Assessment)**

(A = questions to assess Community Efforts; B = questions to assess Community Knowledge of the Efforts; C = Leadership; D = Community Climate; E = Community Knowledge about the issue; F = Resources)

**Introduction to Interview**

Hello, my name is __________ and I come from the FUNSALUD clinic. We are talking and having interviews with key people who live in the communities at El Trifinio to ask about what they know about malnutrition. During this interview, your name, the name of your community and anything that you say will be kept private and not shared with anyone except other members of the research study team.

Before we start the interview, I want to take a few minutes to tell you what I expect from our conversation today:

1. First, I want you to understand that your participation in this interview is completely voluntary. Our priority is your safety and well-being. If at any time you feel the need to end the interview (for example, you find it difficult to answer questions), please let me know.
2. Second, I want to remind you that this discussion is confidential. Your name, the name of your community, and anything you say will be kept private and not shared with anyone except other members of the research team. Your comments will not be associated with your name, and I don't expect you to say anything to me that will make you feel uncomfortable. But what I do expect is that you will be honest with your answers to the questions I will ask you.
3. Third, I want you to know that there are no right or wrong answers, I want to know your honest opinion. Your comments are very important to us. So please, if you have something positive or negative to say, I want to know what you are thinking.

Ok, now I will start with the interview

**DEMOGRAPHICS**

1. Gender: 🞏 MALE 🞏 FEMALE
2. What do you work on? (if applicable)? _______________________________________
3. What is your age range?

🞏 19-24

🞏 25-34

🞏 35-44

🞏 45-54

🞏 55-64

🞏 65 and above

1. What town/community do you live in? _____________________________
2. How long have you lived in El Trifinio? _______________
3. What town/community do you work in? ________________________

**Community Readiness Assessment Interview Questions**

1. Using a scale from 1-10, to what extent do you think malnutrition is a problem in El Trifinio? *(1 being “not a concern at all” and 10 being “a very great concern”)* Please, tell me more (D) (Community Concern)

**DIMENSIONS A & B: COMMUNITY EFFORTS (programs and activities) and COMMUNITY KNOWLEDGE OF EFFORTS**

1. Do you know programs or activities that are being carried out or have been carried out to improve the lack of food and nutrition? (A)

(If any program or activity was mentioned, continue to question 3. If NO program or activity was mentioned, skip to question 10)

1. How long have these efforts, activities or programs been going on in El Trifinio? (A)
2. Do people in the communities use these programs? Why or why not? (A)
3. What are the strengths of these activities or programs to improve food shortages and nutrition? (A)
4. What are the weak points of these activities or programs to improve food shortages and nutrition? (A)
5. Using a scale from 1-10, how informed do you think the people in the community are about programs or activities that seek to reduce or treat malnutrition? *(1 means not at all informed and 10 means very well informed)* Please explain. (B) (Community Awareness)
6. What do people in the communities (including you) know about these programs or activities to reduce malnutrition? (B)
7. What information is available to the people living in El Trifinio about these programs or activities? Do you know if community members use this information? (B)
8. Who in El Trifinio is trying to do something to reduce child malnutrition? If so, please tell me more. (B)

**DIMENSION C: LEADERSHIP**

Now, I’m going to ask you some questions about what people in the community think about child malnutrition.

1. Do you know who at the Trifinio communities believe that malnutrition is an issue that should be addressed? Please, tell me more. (C)
2. What are community leaders or people doing to reduce malnutrition? *(For example, are leaders merely supportive or are they more actively involved? Or, for example, are they involved in a committee, or have they allocated resources to address the issue?)* (C)
3. Would people in the community be interested in new programs or activities to deal with malnutrition? If so, what do you think they would do? (C)

**DIMENSION D: COMMUNITY CLIMATE**

1. Do you think that addressing malnutrition is very important for people in your community? Please, tell me more. (D)
2. Do El Trifinio communities support addressing the problem of malnutrition? If yes, do you think they would actively support or participate in these programs or activities? (D)

**DIMENSION E: KNOWLEDGE ABOUT THE ISSUE**

1. In general, how much do people in the community know about malnutrition? *(e.g. the consequences of malnutrition in children during their growth and development)* (E)
2. How and where do people in your community receive information about the problem of malnutrition? *(e.g. they pass the information by word of mouth, they hear it on the radio, they read it in the newspapers, in brochures, they watch it on television, they see it on Facebook or Instagram, they receive messages on WhatsApp)* (E)
3. Is there data or information on how common malnutrition is in El Trifinio? (E) If so, how do people obtain this information?

**DIMENSION F: RESOURCES FOR EFFORTS (time, money, people, space, etc.)**

1. If a child is suffering from malnutrition in your community, who would you turn for help? Why? (F)
2. What opinion do people in the community and/or local businesses have about being able to support activities or programs that fight malnutrition? If so, tell us more. (F)

**Additional questions:**

1. Who are in charge of improving the nutrition of children in El Trifinio? If no is in charge, who do you think should be in charge? (C)
2. What are the primary barriers or difficulties (or obstacles) to address this issue in the community?
3. How easy or difficult do you think it is to get food in the Trifinio communities? Tell me more, is the food expensive or cheap?
4. Tell me about some meals or dishes that are cooked daily in the homes of your community
   1. Why do you choose this food(s)?
      24.2 Are these meals the same for children and adults? or are they different?
      24.3 Are they the same for men and women?
5. Do you consider that rice (whole grain brown rice and/or polished white rice) is a common food ingredient or widely used by families in this community? Is it white rice or brown rice?
6. Who is the person in charge of purchasing food in the family?
   1. Does your family grow any food? Who is the person in charge of sowing?
7. Do you think that the people in the communities would be interested in using other foods or ingredients for the meals that are cooked or eaten daily (such as porridge, atol or tortillas, or any food that they mention in the question #24)?

**Questions related to malnutrition**

1. How do you define malnutrition?
2. How do you treat a malnourished child?
